# The Age of Artificial Intelligence: Use of Digital Technology in Clinical Nutrition

**DOI:** 10.1007/s40137-021-00297-3

**Published:** 2021-06-08

**Authors:** Berkeley N. Limketkai, Kasuen Mauldin, Natalie Manitius, Laleh Jalilian, Bradley R. Salonen

**Affiliations:** 1 Vatche & Tamar Manoukian Division of Digestive Diseases, UCLA School of Medicine, 100 UCLA Medical Plaza, Suite 345, Los Angeles, CA 90095 USA; 2 Department of Nutrition, Food Science, and Packaging, San José State University, San José, CA USA; 3 Department of Anesthesiology, UCLA School of Medicine, Los Angeles, CA USA; 4 Division of General Internal Medicine, Mayo Clinic, Rochester, MN USA

**Keywords:** Nutrition, Parenteral nutrition, Enteral nutrition, Digital health, Machine learning, Wearables

## Abstract

**Purpose of review:**

Computing advances over the decades have catalyzed the pervasive integration of digital technology in the medical industry, now followed by similar applications for clinical nutrition. This review discusses the implementation of such technologies for nutrition, ranging from the use of mobile apps and wearable technologies to the development of decision support tools for parenteral nutrition and use of telehealth for remote assessment of nutrition.

**Recent findings:**

Mobile applications and wearable technologies have provided opportunities for real-time collection of granular nutrition-related data. Machine learning has allowed for more complex analyses of the increasing volume of data collected. The combination of these tools has also translated into practical clinical applications, such as decision support tools, risk prediction, and diet optimization.

**Summary:**

The state of digital technology for clinical nutrition is still young, although there is much promise for growth and disruption in the future.

## Introduction

Accelerating advances in the power and utilitarian benefit of digital technology have led to its pervasive integration into society. Each step throughout the day—from the moment we awaken to the sound of an alarm to the moment calming music is played before bedtime—digital devices play a central role in these functions. Virtually all industries, including those unrelated to technology, now rely on digital technology in some form to capture data, perform calculations, and automate processes. Nonetheless, beyond the sheer scale of availability and assimilation of such technologies in society is the remarkable computing power within reach. For instance, over 80% of Americans today wield more computing power in their palms than the Apollo 11 guidance computer that first landed Neil Armstrong on the moon [1]. Such advances in processing power and data storage sizes, and an inverse reduction in cost per gigaflop or gigabyte, respectively, have similarly led to development of increasingly sophisticated applications. Storage and analysis of “big data” no longer require supercomputers that occupy large climate-controlled rooms. In the medical industry, immense amounts of clinical and administrative data are routinely collected by health networks to evaluate trends in care delivery, identify areas for cost reduction, and perform outcomes-based research. Artificial intelligence (AI) has been harnessed to help understand complex biological phenomena, diagnose diseases, predict clinical outcomes, and design novel therapeutics. The ubiquity of mobile and smart devices even provide opportunities for more personalized real-time data collection, data synthesis, analysis, and feedback at the consumer to enterprise levels.

Following the rise of the medical technology (“medtech”) industry, there has been a burgeoning interest in the application of similar core technologies for nutrition. These technologies offer the opportunity to optimize nutrition, as information about diet intake, interpretation of the diet in the context of the person and their health, and the ability to generate practical feedback are important features of clinical nutrition. Implementation into clinical practice may range from the use of mobile apps to track diet intake and the use of wearable technologies to collect supportive data to the development of decision support tools for parenteral nutrition and the use of telehealth for remote assessment of nutrition.

## Mobile Applications

Mobile health applications represent an opportunity for increased patient engagement, data gathering, and remote monitoring of outcomes outside of the healthcare facility. There now exist an estimated 165,000 publicly available mobile health apps with wellness management and disease management in leading areas [2]. In 2020, the mobile health market was valued at 40 billion dollars and is expected to grow 17.7% from 2021 to 2028 [3]. Use of mobile apps for monitoring health data is thus a growing area and surveys of mobile phone users in the United States indicate that 58% of mobile phone users have downloaded a health-related app to their device [4]. Among registered dietitians, nearly 83% report use of mobile apps in their practice [5]. Despite widespread use among individuals, apps remain an ongoing area of investigation for its use in healthcare management.

Applications focused on diet and weight loss are widely utilized, and it is estimated that over 10,000 apps are currently available for diet and weight loss [6]. A 2015 systematic review and meta-analysis of studies evaluating mobile apps for weight loss showed a mean reduction of body mass index (BMI) by 0.43 kg/m^2^ among mobile app users, and users may benefit from more continuous feedback on their health interventions [7]. For a healthcare provider, mobile apps may provide further opportunities to assess dietary patterns of patients instead of relying on dietary recalls alone. This feature may provide added value to the nutrition care process, as patients often under-report their intake or engage in recall bias due to factors such as body dissatisfaction or desire for social approval [8].

Diet-focused apps vary widely in terms of their functions and ease of use. Self-monitoring of diet and physical activity are commonly integrated features and individuals may record their dietary intake and physical activity, while establishing goals to meet in these areas, thereby receiving continuous data feedback on their behavior [•^9^]. Apps may also be purveyors of health information, including tips on weight management or diet, whether or not this has been vetted or verified by healthcare professionals and experts. Other apps may have a component of social engagement with the ability to interact on group forums or connect with other users. Features among nutrition apps vary widely, yet all share some commonality in terms of tracking the user’s day-to-day dietary intake and physical activity. Examples of applications in the weight loss space employing such features are shown in Table 1.

**Table 1:** Some mobile applications for weight loss management

Mobile app	Features
Cronometer	• Fasting timer—function to track fasting and see effect of fasting over time • Food tracker—includes ability to input foods based on scanned bar code, filter food search tool based on brands, restaurants, etc. Uses multiple databases to integrate nutritional data—Nutrition Coordinating Center Food & Nutrient Database through the University of Minnesota (NCCDB), United States Department of Agriculture National Nutrient Database for Standard Reference, The Canadian Nutrient File (CNF 2015), Irish Food Composition Database (IFCDB), The Dutch Food Composition Database (NEVO), McCance and Widdowson’s The Composition of Foods Integrated Database (United Kingdom food supply), Australian Food Composition Database (NUTTAB). Nutrition daily report includes calories consumed, macronutrient calories and percentages, protein by amino acid composition, fat by composition including omega-3 and omega-6, calories burned, basal metabolic rate, fiber intake, micronutrients including vitamin A, potassium, vitamin d, vitamin E, vitamin K, B vitamins (B1, B2, B3, B5, B6, B12, folate), minerals (calcium, copper, iron, magnesium, manganese, phosphorus, potassium, selenium, sodium, zinc) • Healthcare Professional Accounts—health privacy security standards. Ability to view and edit patient nutrition targets, graph and print reports, restrict client accounts (i.e., limit access to calorie information for a patient with an eating disorder), find new clients, and become an affiliate. At time of print, $24.95 billed monthly • Reports and charts—ability to track changes in calories consumed and weight changes. Premium member features include weight and body fat, lipid panel, blood pressure + heart rate
MyFitnessPal	• Food tracker—ability to adjust calories and macronutrients based on individual needs and ability to share diary with friends on the app. Healthcare providers can create an account on the app but do not have a special designation in the app • Food database tool for determining nutritional content includes carbohydrates, fats (including grams of fat from polyunsaturated, monounsaturated, saturated, trans fats), cholesterol, sodium, potassium, carbohydrates, sugars, protein, vitamin A, vitamin C, calcium, iron • Exercise—ability to input exercise by type and duration, synchronize data with wearable devices, and update nutritional needs accordingly • Report generation—trends in weight, calories, exercise minutes, calories burned, macronutrients, fiber, micronutrients • Community forums—members can create conversation threads or add friends on the app
Noom	• Use of psychology and behavior change to implement weight loss—intake questions include screening questions for previous interventions tried, risk factors, motivation, and barriers. It includes daily articles and challenges, unlimited access to a personal coach approved by the National Consortium for Credentialing Health and Wellness Coaches, tools to track and monitor progress, support group of peers, biometric tracking including blood pressure and blood glucose). At time of writing, it costs $59 per month • Research—A 2016 retrospective cohort study of nearly 36,000 adults utilizing the NOOM app demonstrated weight loss among 77% of app user (mean duration of app use—267 days). Analyzed variables that contributed to weight loss include gender, baseline body mass index, weight input frequency, exercise, and dinner input frequency (*p* < 0.001)

*NCCDB* nutrition coordinating center food & nutrient database; *CNF* Canadian nutrient file; *IFCDB* Irish food composition database; *NEVO* Dutch food composition database; *NUTTAB* Australian food composition database

Another area of interest in mobile app nutrition includes those catered to specific disease states. Apps for diabetes, for example, may provide users with a better understanding of how their blood sugar management relates to their diet and behavior. While a number of applications exist, a 2018 technical brief by the Agency for Healthcare Research and Quality evaluated currently available mobile apps for diabetes self-management and found that among hundreds of apps for diabetes, studies showed that only 5 were associated with clinically meaningful improvements in biomarkers such as hemoglobin A1c (HgbA1c) [10]. The report concluded that more longitudinal, high-quality studies are needed. As diabetes represents but one of many disease states that may benefit from mobile app use, rigorous studies evaluating outcomes in app use for disease management are often outpaced by the development and widespread use of the apps. For patients with diabetes, apps such as Day Two, Glucose Buddy, and Dario Health may be utilized (Table 2). In addition to diabetes, gastrointestinal disease is another area where mobile apps may play a role in monitoring and management. Some of the applications in this area are shown in Table 3.

**Table 2:** Some mobile applications for diabetes management

Mobile app	Features
Day Two	• Utilizes the Algorithm Diet—requires that patients submit a stool sample to establish a profile of the patient’s gut microbial ecosystem; integrates this with lab values such as HgbA1c to determine individualized dietary plans for patients. Provides insights into how body uniquely metabolizes foods in order to utilize food as medicine approach to managing type 2 diabetes
Glucose Buddy	• Glucose tracking—tracking of blood sugar, medication, hemoglobin A1c, weight, and blood pressure • Dietary intake—ability to track intake via photo recognition or scanning product bar codes • Data export—download reports to share with healthcare providers • Education and coaching—12-week diabetes education plan with 5 min lessons, ability to meet one-on-one with certified diabetes coaches • Integration with other wearable devices—integrate data from FitBit, Apple Watch, etc
Dario Health	• Blood glucose monitoring system and blood pressure monitoring system—Dario Meter combines meter, lancet, and test strips in a unit to enable blood glucose testing and data upload within 6 s of applying drop of blood to the test strip. Per Dario app information page, tested to meet FDA guidance for accuracy, with 95% of measurements within 15% of true lab-tested value • Hypo alert with global positioning system (GPS) locator—If blood glucose monitor reads dangerously low blood glucose levels, app will text message up to 4 emergency contacts with GPS information • Diet tracking—tag foods eaten and application will calculate carbohydrates. Ability to share data with healthcare providers

*FDA* food and drug administration; *GPS* global positioning system

**Table 3:** Some mobile applications for gastrointestinal conditions

Mobile App	Features
Cara Care: IBS	• IBS, FODMAP tracker—tracks bowel movements, stress, dietary intake, pain, and other GI symptoms; shows trends and correlations among inputted data and symptom outcomes • 12-week IBS program—self-paced low FODMAP diet plan with access to tips for managing IBS based on science, medically validated IBS questionnaires, and chat with dietitian • Data sharing—ability to share data with medical provider
Gali Health: IBD	• Artificial intelligence health assistance—gathers data on date of diagnosis, disease form and activity, symptoms, and challenges with current care in order to create personalized recommendations • Health tracking and analytics—daily health and treatment monitors, ability to generate reports and share with care team • Information—chat feature with Gali for asking IBD-related questions, gather disease-related insights • Community—ability to comment on posts, ask questions, share experiences with users
My Symptoms	• Food and symptom diary—ability to track food, symptoms, bowel movements, exercise, stress, sleep, in order to help determine patterns and trigger foods in the diet • Reports—provides diary analysis to illustrate correlations between various factors and symptoms

*FODMAP* fermentable oligo-, di-, monosaccharides and polyols, *IBD* inflammatory bowel disease, *IBS* irritable bowel syndrome

Given the multitude of mobile health apps available and widespread use among individuals, clinicians may benefit from employing these tools in their practices. While many barriers currently exist related to collection and use of personal data (e.g., assurance of accuracy privacy concerns, regulatory oversight, or lack of integration into the healthcare system) [2], these challenges may be addressed through future collaboration with research and healthcare institutions. While mobile apps may be an imperfect process at this time, they still provide value to patients looking to utilize more data and engagement for improving health outcomes.

## Wearable Technologies

Another emerging technology in health care is wearable devices. While a variety of consumer health monitoring devices relevant to clinical nutrition assessment exists, this section will focus on noninvasive wearable technologies, which are defined as compact devices that present information to users, enable user interactions, and are meant to be worn on the body [11]. Wearable devices relevant to clinical nutrition care discussed here focus on the widely used smartwatches, the more experimental wearable devices for dietary assessment, and emerging wearable device technologies.

### Smartwatches for Nutrition Assessment

Smartwatches are by far the most popular wearable health monitoring devices [12]. Smartwatch technologies have rapidly advanced in the past ten years, initially starting with simple pedometers to current devices with increasing health monitoring capabilities [13]. Smartwatches for health-care monitoring tend to utilize one or often a combination of the following technologies: accelerometer, pedometer, gyroscope, heart rate, electrocardiography (ECG), pulse oximetry, altimeter, barometer, proximity, microphone, camera, compass, global positioning system (GPS), and/or long-term evolution (LTE) communication [14]. Smartwatches may have started out as standalone devices, but given the greater capabilities (e.g., higher computing power and connectivity) of smartphones, the current smartwatches are meant to be paired with mobile applications to enhance the user experience. Table 4 provides a list of the features of the current popular smartwatches that are relevant to clinical nutrition care.

**Table 4:** Features of current popular smartwatches relevant to clinical nutrition care

Device, cost^a^	Health monitoring features	Additional features
Apple Watch Series 6	Tracks fitness (distance, elevation), monitors heart rate, monitors blood oxygen levels, takes ECG, tracks sleep	Can customize digital watch face Water resistant 50 m, GPS, maps, plays music, Apple pay, can be used to call and text when smartphone is nearby
Fitbit Sense	Tracks fitness (steps, distance, elevation), monitors heart rate, monitors blood oxygen levels, takes ECG, monitors on-wrist skin temperature, tracks heart rate variability, tracks sleep, estimates daily stress score, can be used to track menstrual health	Can customize digital watch face Water resistant 50 m, GPS, plays music, Fitbit Pay, can make calls and text when smartphone is nearby
Samsung Galaxy Watch3	Tracks fitness (distance), monitors heart rate, monitors blood oxygen levels, takes ECG, tracks sleep, monitors stress levels, fall detection, analyzes running movement and suggests stride improvements	Can customize watch design at purchase GPS, plays music, and email, text messaging notifications and calls when smartphone is nearby
Wear OS Google Smartwatch software	Tracks fitness (steps, distance), monitors heart rate	Variety of designs: Google partnered with watch designer brands GPS, Maps, plays music, Google Pay, Google calendar synching, and email, text messaging notifications and calls when smartphone is nearby

^a^Websites accessed in January 2021

Smartwatches allow patients to passively gather data about their activities of daily living. These patient-generated health data can then be shared with healthcare providers. A recent qualitative study found that healthcare providers valued patient smartwatch data, because these data can be used to initiate productive discussions and inform patient care decisions [15]. Smartwatches can be used to gather baseline data and track patient progress and efficacy of interventions. For example, a decline in functional status is one of the recommended criteria for the identification and documentation of malnutrition [16]. Activity trackers can help clinicians monitor and evaluate any trends in functional decline or improvement through tracking activities of daily living. Of note, smartwatches have been shown to more effectively recognize hand-based activities (e.g., eating, typing, playing catch, etc.) when compared to smartphones, but specific activity tracking recognition technology still requires much fine tuning [17]. Using machine learning approaches, future development work can expand to recognition of specific activities with more accuracy.

The most recent technology advance in smartwatches is the addition of cardiovascular health measures. As shown in Table 4, popular smartwatches include features to monitor heart rate, monitor blood oxygen levels, take ECG, and/or track heart rate variability. In 2017, the Apple Watch accessory wristband, the Kardia Band, was the first to receive Food and Drug Administration (FDA) clearance for detection of atrial fibrillation, the most commonly encountered arrhythmia in clinical practice [18, 19]. Since then, Apple Watches have evolved to include inherent ECG monitoring capabilities with the newer series watches (Series 4 and higher) not requiring an accessory wristband to monitor and detect atrial fibrillations [18–20]. Primary research indicates direct-to-consumer smartwatches that passively detect atrial fibrillations can be useful in clinical settings. However, the algorithms used for specific and sensitive detection of arrhythmias will need to be further validated [21, 22]. Overall, cardiovascular health data provided by smartwatches can be used by nutrition care providers to integrate into their nutritional assessments to monitor and evaluate the efficacy of nutrition interventions for patients.

### Wearable Devices for Dietary Assessment

Currently, much of dietary self-assessment occurs via mobile applications (discussed in section “Mobile Applications”) that allow users to actively maintain digital dietary records. Patients often show their digital food diaries to clinicians during nutrition visits. Despite still being in the prototype stages and with limited utility in the clinical setting, wearable dietary monitors are being developed as a new method to passively capture dietary intake [23]. The most promising wearable dietary intake sensors currently being researched are sound, image, and/or motion [23].

Acoustic-based food intake wearable devices utilize microphones to detect chewing and/or swallowing patterns that could theoretically give insight into the type and/or relative quantity of food being eaten. For example, in a study using a tiny microphone embedded in an ear device, researchers created a sound-based recognition system that was able to distinguish between three test foods (potato chips, lettuce, and apple) with 94% accuracy [24]. The system analyzed acoustic variables (structural and timing) associated with chewing and used this data to predict bite weight, defined as “a quantity of food amount that is ingested into the mouth with each bite taken.” Bite weight prediction models were selected based on recognized food types. While this study was limited in the types of foods detected, one can imagine that the employment of machine learning can be used to advance this field of sound-based dietary intake sensors in food intake tracking.

Image-based food intake wearable devices use cameras to classify foods and/or estimate portion sizes. One notable wearable image-based device is the eButton, which is a miniature computer with a camera embedded in a 6 cm (or 2.4 in) diameter button, meant to be worn on the chest [23, 25–28]. The eButton automatically takes images at a preset rate of a meal being eaten and theoretically, the images can be analyzed by an algorithm to detect the food item and portion size based on environmental cues such as plate and eating utensils [28].With food item and portion size information, the calories and nutrients data can subsequently be obtained from a linked dietary database. Other wearable devices that capture digital images in dietary assessment typically require subsequent coding by nutritionists to identify the type and amount of foods eaten [26]. With smartphones being more commonplace, patients may opt to use mobile applications that utilize the camera on their smartphones to take food images for dietary assessment [25], although using smartphones for dietary assessment would require active rather than the passive data collection offered by wearable devices.

Motion-based dietary assessment wearable devices are often worn on the wrist to track wrist movements during eating. These devices integrate an accelerometer and/or a gyroscope to record lifting, turning, and/or rotation movements of the wrist to count bites as a proxy marker for caloric intake [23, 27]. As these devices are meant to be worn all day, the sensor needs to be able to distinguish between eating events and non-eating wrist movements. Researchers have tested devices that are able to pick out periods of eating during all-day tracking with good accuracy [29]. Algorithms to estimate number of bites using inertial data and predictive equations to estimate caloric intake associated with a single bite have been developed [30, 31]. It should be noted that these devices need to be worn on the dominant hand used for eating.

Importantly, these wearable dietary intake sensors are still in development. Future work will need to focus on algorithms that can distinguish between different foods, especially solid versus liquid foods, more accurately estimate food portions and volumes, and remove background sound, image, and motion data in real-world environments that are not related to food ingestion. It has been noted that fusion devices that track sound, image, and motion could be developed and coupled with smartphone applications via Bluetooth technology to allow higher computing power and a more sophisticated user interface [28, 32]. In fact, one can imagine adding the detection of eating events and estimation of caloric intake from activity data to current smartwatch features.

### Future Direction of Wearable Devices

Recently released wearable devices reveal the trajectory of the industry. One challenge unique to wearable devices is that device size can limit computing power and battery life. Ideally, wearable devices should be lightweight, comfortable to wear, and power efficient. The trend in pairing wearable devices with smartphone applications is a solution to creating a small, easy-to-wear device with increasing monitoring capabilities. Given that smartphones are ubiquitous nowadays, new and future wearable devices will likely rely on mobile applications for user interface.

As an example, the Amazon Halo wristband health monitor was released in Fall 2020 and there is no screen on the device [33]. The Amazon Halo wristband not only tracks activity (intensity and duration and sedentary time) but also monitors sleep, tracks heart rate, estimates body composition, and detects mood (via tone of voice analysis). The use of the Amazon Halo and its features does require a smartphone and a subscription membership ($3.99/month) with Amazon. The most novel and controversial aspects of the Amazon Halo are its ability to estimate percent body fat from a three-dimensional model of the user’s body based on photos and its tone analysis aimed to help the user communicate more effectively with others [34]. The body composition estimation feature is of interest in nutrition assessment. The mobile application associated with the Amazon Halo creates a three-dimensional model of the user and allows the user to simulate what they may look like with more or less body fat. This wearable device has not yet been studied in clinical trials, so the accuracy of the body composition analyses when compared to other clinical methods (such as bioelectrical impedance analysis and whole-body dual-energy X-ray absorptiometry) remains to be seen. Healthcare concerns have also been raised, especially related to potential body dysmorphia and particularly in adolescent populations, as a result of this body composition feature. Another concern is the clinically appropriate interpretation of the body composition results [35]. While many reference ranges for body fat percentages have been published for various populations, there is no consensus on what is considered normal body fat percentage. There is no one-size-fits-all algorithm to appropriately interpret body composition results. Future algorithm development will need to take into account as many factors as possible (e.g., genetics, ethnicity, fitness, dietary intake, etc.) to present results in the context of overall personalized health.

Another emerging technology in wearable devices is the goal of creating a noninvasive blood glucose monitor. At the January 2021 Consumer Electronic Show (CES) virtual conference, a Japanese startup company, Quantum Operation Inc., showcased a prototype noninvasive glucose monitor [36]. The Quantum Operation Inc. Glucose Monitor looks like a smartwatch and houses a small spectrometer used to scan the blood through the skin for glucose concentrations [37]. The company supplied a sampling of data comparing their monitor’s blood glucose measurements with those taken using a commercial glucometer, the FreeStyle Libre [36]. The sampling data shows variation between the data collected by the Quantum Operation glucose monitor and continuous blood glucose measurements.

As wearable devices move from the realm of wellness monitoring to become more medical devices (where information obtained from devices will be used to make medical decisions), they will be subject to regulation by the FDA [•^13^]. In anticipation, the FDA has been working with wearable device manufacturers, such as Apple, and introduced the Digital Health Software Precertification (Pre-Cert) Program for low-risk device approval [38]. The purpose of the FDA Pre-Cert Program is to “provide more streamlined and efficient regulatory oversight of software-based medical devices developed by manufacturers who have demonstrated a robust culture of quality and organizational excellence, and who are committed to monitoring real-world performance of their products once they reach the U.S. market.”

A recent review of scientific literature in the field of wearable health monitoring technologies revealed the top concerns regarding wearable devices are security and safety, especially if the data from wearable devices are used for medical decisions [39]. A major advantage of using wearable devices for assessing health is the fact that this technology does not rely on subjective information and removes the burden of self-reporting. Wearables can be helpful in clinical interventions as they provide real-time information to users and have the potential to change immediate behaviors. Ideally, a wearable device should transmit real-time data to a patient’s healthcare team, and clinical interventions would be determined based on that data. Moving forward, in order for consumer-based wearable devices to be more widely used in clinical practice, there needs to be ways for user data to be privately and securely shared with clinicians. Ultimately, clinicians will play an integral role in interpretation of wearable device monitoring data, as “data must be interpreted before they can be considered real information” [40]. It is important for clinicians to be familiar with current and emerging wearable devices and their limitations as they help patients transform collected data into useful information for making healthcare decisions.

## Artificial Intelligence

AI is broadly defined as the application of computers to independently or semi-independently perform functions that mimic human intellect. Machine learning, a subset of AI, involves computer algorithms that process and learn from data without requiring explicit programming to define each step. Deep learning is a further subset of AI and machine learning that involves the use of multi-layered neural networks (“deep learning”) to permit far more complex analyses. As suggested in the term, neural networks were designed to mimic the complex neuronal network architecture of the human brain. Similar to how a person learns principles through repeated observations, large datasets are typically required to train the AI system how to interpret data. The AI system can nonetheless improve (“learn”) over time as it gets exposed to more data. Training can be performed in a supervised, semi-supervised, or unsupervised manner. Supervised learning involves the use of labeled data that “teach” the system about the meaning and relationships within the data, such as labeling fruit images with their respective identifiers (e.g., apple, orange, lemon) to allow the system to identify shared and unique features inherent in each fruit type. Unsupervised learning involves the processing of data without human intervention, such as pattern recognition of unlabeled data for clustering, anomaly detection, or reduction of complex data. Basic machine learning functions can include pattern detection, prediction, classification, language processing, and image recognition. These functions can be seen in consumer-oriented services, such as spam filters, real estate price estimators, video recommender systems, chatbots, and facial recognition. These functions can similarly be ported to applications in nutrition. Some domains include diet optimization, food image recognition, risk prediction, and diet pattern analysis.

### Diet Optimization

In an early demonstration of the utility of machine learning for personalized nutrition, Israeli investigators collected one-week data on the diet, anthropometrics, blood parameters (e.g., blood glucose, hemoglobin A1c, cholesterol), lifestyle (e.g., physical activity, sleep), and the gut microbiota in a cohort of 900 healthy individuals [41]. When applying the “carbohydrate counting” approach to estimating post-prandial glycemic response (PPGR), the correlation between a meal’s carbohydrate content and PPGR was statistically significant but modest (*R* = 0.38). The machine learning model that considered the participant-specific data was ,however, more effective at predicting PPGR in both the training (*n* = 800; *R* = 0.68) and validation (*n* = 100; *R* = 0.70) cohorts. To demonstrate the use of PPGR prediction for diet interventions, the investigators conducted a trial on 26 healthy participants who first received instructions on a dietitian-designed diet based on their individual dietary preferences and constraints. Participants underwent another week of diet, blood, and activity profiling while on their custom diets, similarly as the original 900 participants. The 26 participants were then randomized to either the “prediction arm” or the “expert arm.” In the former arm, the machine learning algorithm was used to design a “good” diet composed of low-predicted PPGR and a “bad” diet composed of high-predicted PPGR specific to the individual. In the latter arm, clinical experts designed the “good” and “bad” diets based on prior knowledge of foods with high glycemic burden. Participants would consume both “good” and “bad” diets in independent weeks. For the prediction arm, 83% (10/12) of participants had significantly higher PPGR when consuming the “bad” diet than the “good” diet. For the expert arm, similar trends were observed in 57% (8/14) of participants. This technology has since been commercialized with the Day Two mobile application on the front (Table 2).

### Food Image Recognition

Image recognition is a popular function of deep learning. In health care, deep learning has been used for analyzing radiographic images for diagnosis of pneumonia, dermal images for identification of melanoma, and endoscopic images for detection of colonic polyps [42–44]. For nutrition, a natural use of deep learning would be food image recognition. Early models that trained with 50,000 food images could reach a reasonable accuracy of 78 to 92% for identifying the pre-categorized food image [45]. The top-5 accuracy (where the computer provides its top 5 guesses) was even higher at 91 to 98% accuracy. While these models prove the feasibility for AI to detect food images with reasonable accuracy, a primary limitation is the artificial method to train and test the system. That is, the training dataset would include a finite number of labeled food items and the testing or validation datasets would include the same catalog of labeled food items. However, in a real-world scenario, there are innumerable types of food items. With an early neural network model developed by our laboratory, we were able to achieve similar training and validation performance as the other models, while using 222,285 curated images representing 131 pre-defined food categories [46]. However, in a prospective analysis of real-world food items consumed in the general population, the accuracy plummeted to 0.26 and 0.49, respectfully. Future refinement of AI for food image recognition would, therefore, benefit on training models with a significantly broader diversity of food items that may have to be adapted to specific cultures.

### Risk Prediction

Conventional approaches for analyzing data, such as visualization for trends or use of multivariable regression models, often suffice. On the other hand, the advantage of machine learning is its ability to parse large high-dimensional data to identify complex patterns that would otherwise have been hidden. In an analysis of six waves of the National Health and Nutrition Examination Survey (NHANES) and the National Death Index, investigators compared the ability of Cox proportional hazards and machine learning to predict 10-year cardiovascular disease-related mortality in 29,390 individuals [•^47^]. The Cox proportional hazards model that included age, sex, black race, hispanic ethnicity, total cholesterol, high-density lipoprotein cholesterol, systolic blood pressure, antihypertensive medication, diabetes, and tobacco use appeared to significantly overestimate risk. The addition of dietary indices did not change model performance, while the addition of 24-h diet recall worsened performance. By contrast, the machine learning algorithms had superior performance than all Cox models.

4.4 In a prospective study conducted by the University of Athens (Greece), 2583 participants completed a baseline questionnaire, dietary evaluation, and 10-year follow-up [48]. The dietary instrument was the European Prospective Investigation into Cancer and Nutrition (EPIC)-Greek questionnaire. Multiple linear regression and machine learning were compared in their ability to predict the 10-year Cardiometabolic Health Score (a composite of cardiovascular disease, diabetes mellitus, hypertension, and hyperlipidemia). The linear regression models had a predictive accuracy of 16 to 22% when classifying the dietary pattern within the same tertile as the Cardiometabolic Health Score; the machine learning models had a higher accuracy around 40%.

### Diet Pattern Analysis

In a prospective study of 7572 pregnant women in the Nulliparous Pregnancy Outcomes Study: monitoring mothers to be (nuMoM2b), participants completed the Block 2005 Food Frequency Questionnaire to reflect their typical dietary intake around 3 months prior to conception [49]. The investigators compared multivariable logistic regression with machine learning to estimate the risk of adverse pregnancy outcomes based on fruit and vegetable consumption. Among women in the ≥ 80th percentile of total fruit or vegetable density consumption, there was a modestly lower incidence of preterm birth, small-for-gestational-age births, gestational diabetes, or pre-eclampsia. The logistic regression model did not identify an association between fruit and vegetable consumption and adverse pregnancy outcomes, while the machine learning model found that the highest fruit or vegetable consumers had lower risk of preterm birth, small-for-gestational-age birth, and pre-eclampsia.

## Digital Technology and Nutrition Support

The nutrition support team (NST) is a specialized team that provides expertise and guidance to medical teams on the nutritional needs of patients [50]. NST members vary across institutions but may be compromised of physicians, advanced practice providers, dieticians, nurses, and pharmacists. Digital innovations, improvements, and integrations into the electronic health record (EHR) have impacted nutrition care provided by the NST over a spectrum of activities, such as diagnosis and coding, treatment interventions, and follow-up care [51].

Since the NST is typically a consultative service, an important first step is the recognition of a nutritional concern, such as malnutrition, by the primary team [52]. Malnutrition is a clinical condition where the patient is undernourished and not meeting their nutritional needs [52, 53]. Recognition of malnutrition is important because it is associated with increased morbidity and mortality [52, 54]. Numerous validated malnutrition screening tools (e.g., NRS-2002, MUST, SGA) have been created and can be utilized for screening [52]. Importantly, the widespread adoption of EHRs has enabled the integration of these screening tools into clinical workflows, such as admission orders or office visit intakes. In addition, the use of embedded screening tools in the EHR allows the ability to capture the discrete data and can drive clinical decision support processes that can be configured to notify the NST to perform a formal malnutrition assessment [51, 55]. If malnutrition is not initially present, patients can be rescreened at regular intervals for ongoing assessment during the course of longitudinal care of the patient. One of the strengths of the EHR is the ability to capture data for trending and activation of alerts if clinical decision support logic is met.

After a positive malnutrition screen, the NST performs a nutrition assessment of the patient to determine if the patient meets criteria for malnutrition. The nutrition assessment includes food intake and nutrition history, anthropometrics, nutrition focused physical exam, and review of clinical and medical history as well as review of tests and procedures [51]. A study by McCamley et al. demonstrated that after implementation of an EHR, there was not only a 72% increase in admissions requiring nutrition intervention but also the time spent per nutrition event was reduced by 22% post-EHR implementation. If after the nutrition assessment by the NST, the patient meets clinical criteria for malnutrition, the NST member or clinician should then accurately document and code the malnutrition diagnosis. Importantly, this has implications that not only can affect patient care but also is important for appropriate coding, billing, and reimbursement [51, 56, 57].

For patients unable to meet their nutritional needs through enteral means alone, parenteral nutrition (PN) is a nutritional modality that can be utilized by the NST to meet the patient’s nutritional needs through intravenous rather than enteral route. PN is a complex admixture that contains both macro- and micronutrients along with electrolytes and trace elements. Although PN can be lifesaving and life-sustaining, it is a high-risk treatment that has the potential to harm patients if ordered or administered incorrectly [58]. In order to reduce the chance of error, PN is recommended to be ordered through a computerized provider order entry system (CPOE) [59]. By utilizing CPOE, alerts can be developed to notify the prescriber if doses exceed the recommended or safe clinical limits or exceed the limits of compatibility. After entry of the PN order, another digital advance is the integration of the EHR to the automated compounding device (ACD) that prepares the PN. The digital link from the order to the ACD eliminates any manual transcription, including handwritten, verbal, or fax transmissions in the PN workflow thereby reducing the chance for error and harm to patients [58].The supporting evidence for this workflow was highlighted in a 2016 study from a large academic pediatric hospital where the frequency of PN errors was 0.27% (230 errors/84,503 PN prescriptions) with no errors due to transcription [60].

Like PN, enteral nutrition (EN) has also benefited from the digital advances in health care. The number of EN products available on the market today is large, and hospitals often also have a number of these products available on their formulary to order for patients. In addition, capturing the EN product used as well as documenting the amount used is important for the overall care of patients. To address these issues, Kamel et al. demonstrated the steps for development of an electronic nutrition administration record (ENAR) with a linked nutrition tab in their EHR [61]. Working with the EHR vendor, they were able to create order panels that standardized the EN ordering process. After EN administration, the amount of EN was documented and captured for inclusion in the patient’s ins and outs, which is important for ongoing clinical volume assessment.

Lastly, the coronavirus disease 2019 (COVID-19) pandemic brought about significant changes in healthcare delivery [62]. NSTs were not spared as from the system-wide disruption and were required to adapt how they practiced nutrition care [63]. One significant advancement was the use of a virtual care model [62–65]. In this type of model, the patients and the care team do not need to be physically co-located and the patient is not seen in a traditional face-to-face visit. The hospital or clinic visit is instead conducted through a virtual format such as video, telephone, or electronic consultation. The significance of the virtual visit is that it allows the continued involvement of specialized teams, such as an NST, to be involved in the patient care with lowered risk of infection transmission for providers and patients. Meyer et al. published their experience with creation and experience with virtual NST model in a multisite healthcare system [•^66^]. With implementation of the virtual NST, they demonstrated improved appropriateness of PN use (97.2% vs 58.9%) and also improved glycemic control (83.5% vs 62.2%).

Keating et al. published their work assessing the agreement and reliability between clinician-measured and patient self-measured clinical and functional assessments for use in remote monitoring, in a home-based setting, using telehealth [67]. They noted that patient self-assessed clinical and functional outcome measures for metabolic health and fitness had good agreement and reliability on average with face-to-face clinician-assessed outcome measures, but that aside from body weight, no clinical or functional outcome was deemed acceptable when compared with minimal clinically important difference. As health systems increasingly develop hybrid care pathways incorporating both in-person and remote nutritional assessments, there is an increasing need for the development of standardized measures for remote nutritional assessments [•^68^]. Additionally, development of virtual care pathways will need to consider patient training to improve the uptake and reliability of patient home-based health assessments and anthropometric measurements.

## Conclusion

The adoption of digital devices and AI have opened exciting avenues for personalized nutrition and optimization of nutrition care. Mobile applications and wearable technologies have since facilitated longitudinal, real-time, and multi-type data collection, while advances in computing power and refinements in machine learning algorithms have permitted high-dimensional analyses of large datasets to generate meaningful observations. The multi-modal integration of technology has, thus, allowed for development of sophisticated applications in medicine and nutrition. Similarly, digital health has improved the quality and safety of nutrition support care, while telehealth helped preserve this quality of care during the COVID-19 pandemic. As the application of cutting-edge digital technologies lags in nutrition relative to the medical or other consumer-oriented industries, disruptive technologies in nutrition are still forthcoming but near. As such, continued research and development in these areas will indubitably produce technological innovations for nutrition that would have once been relegated to science fiction just a few years ago.
